# Economic burden and quality of life of caregivers of patients with sickle cell disease in the United Kingdom and France: a cross-sectional study

**DOI:** 10.1186/s41687-024-00784-y

**Published:** 2024-09-26

**Authors:** Martin Besser, Sian Bissell O’Sullivan, Siobhan Bourke, Louise Longworth, Giovanna Tedesco Barcelos, Yemi Oluboyede

**Affiliations:** 1 Department Haematology, Addenbrooke’s Hospital, Cambridge, UK; 2 Putnam PHMR, Ceva House, Excelsior Road, Ashby-de-la-Zouch, LE65 1NG UK; 3 HTA Value and Evidence, Sickle Cell Disease, Pfizer AG, Zürich, Switzerland

**Keywords:** Sickle cell disease, SCD, Caregiver burden, Productivity loss, Health-related quality of life, HRQoL

## Abstract

**Background:**

Sickle cell disease (SCD), a genetic blood disorder that affects red blood cells and oxygen delivery to body tissues, is characterized by haemolytic anaemia, pain episodes, fatigue, and end-organ damage with acute and chronic dimensions. Caring for patients with SCD imposes a high burden on informal caregivers. This study aims to capture the impact on health-related quality of life (HRQoL) and economic burden of caregiving for patients with SCD.

**Methods:**

Validated instruments of HRQoL (EQ-5D-5L, Carer Quality of Life-7 dimensions [CarerQol-7D]) and productivity (Work Productivity and Activity Impairment Questionnaire: Specific Health Problem [WPAI: SHP]) were administered via a cross-sectional online survey to caregivers in the United Kingdom (UK) and France. Demographics, HRQoL, and economic burden data were analyzed using descriptive statistics. Economic burden was determined using country-specific minimum and average wage values. Subgroup analysis examined caregivers with and without SCD.

**Results:**

Sixty-nine caregivers were recruited (UK, 43; France, 26), 83% were female, and 22% had SCD themselves. The mean (SD) caregiver EQ-5D-5L score was 0.66 (0.28) (UK, 0.62; France, 0.73), and the mean CarerQol-7D score was 80.69 (24.40) (UK, 78.72 [25.79]; France, 83.97 [22.01]). Mental health problems were reported in 72% and 70% of caregivers measured using the EQ-5D-5L and CarerQol-7D, respectively. Financial problems were reported by 68% of caregivers, with mean annual minimum wage productivity losses of £4209 and €3485, increasing to £5391 and €9319 for average wages. Sensitivity analysis determined additional HRQoL decrements for caregivers with and without, SCD.

**Conclusion:**

Caring for patients with SCD impacts the HRQoL and economic burden of caregivers. Further research to support the complex needs of SCD caregivers is required.

**Supplementary Information:**

The online version contains supplementary material available at 10.1186/s41687-024-00784-y.

## Abstract (plain language summary)

Caregivers play an important role in the lives of the people with sickle cell disease that they care for; however, their mental and physical health and their finances can be affected, particularly if their ability to work is impacted. The extent to which caring for a person with sickle cell disease impacts caregivers is not fully understood. In this study, 69 caregivers of a family member, partner, or friend with sickle cell disease in the United Kingdom or France completed an online survey to share their experiences about how caring for someone with sickle cell disease can impact a caregiver’s quality of life and financial well-being. Caregiving negatively affected the quality of life of caregivers compared with people in the general population and caused a large financial and social burden. Around 70% of caregivers reported having mental health problems, 68% reported financial problems, and lost work hours and lost income were not uncommon. More research is needed to understand the specific needs of caregivers of people with sickle cell disease and how best to support them.

**Supplementary Information:**

The online version contains supplementary material available at 10.1186/s41687-024-00784-y.

## Introduction

Sickle cell disease (SCD) is a group of inherited multisystem blood disorders caused by a mutation in the hemoglobin beta chain that leads to the formation of “sickle”-shaped red blood cells (RBCs) rather than normal, flexible, disk-shaped ones [1]. Sickled RBCs are incapable of properly delivering oxygen throughout the body and are more likely to rupture prematurely, leading to haemolytic anaemia and vaso-occlusive crisis (VOC). Crises occur when sickled RBCs block or decrease blood flow to the extent that tissues become deprived of oxygen, causing severe, painful episodes [2]. Patients with SCD experience fatigue and progressive multiorgan damage, including chronic kidney disease, cardiovascular disease, cerebrovascular complications, and pulmonary hypertension. SCD is the most common monogenic disorder worldwide; it affects approximately 1 in 2000 births in the United Kingdom (UK) and 1 in 500 births in France [3, 4]. SCD is linked with decreased quality of life (QoL), significant morbidity, increased mortality, healthcare resource utilization, and associated costs [5].

SCD disproportionately affects individuals of lower socioeconomic status, amplifying existing inequalities [6]. Moreover, employees with SCD can experience a drastically lowered lifetime earning potential in the workplace compared with those without SCD [7]. Consequentially, informal caregivers may need to compensate for such financial shortcomings. Given the long-term complications of SCD, informal caregivers of patients with SCD can experience substantial emotional and financial burden over time [8]. Beyond the direct financial implications, providing at-home care to someone with SCD can affect the productivity of caregivers. To date, the literature investigating the financial, mental, and physical health effects of SCD on caregivers is limited, particularly for those living in the UK and France.

Caregivers play a critical role in managing the complex and chronic nature of the SCD, yet the impact of this on HRQoL and the economic burden remains underexplored. The aim of this study was to assess the health-related QoL (HRQoL) and financial burden borne by informal caregivers of patients with SCD in the UK and France. By addressing this gap in the research, our study aims to provide a comprehensive understanding of the caregiving experience for individuals with SCD.

## Methods

An online cross-sectional survey was created and administered in both English and French for a 6-week period from March 2022. It included sociodemographic variables that influence the caregiver/care-recipient burden such as age, gender, number of patients with SCD that the caregiver cared for (when multiple members of the same household were care recipients, demographic data were collected for the youngest care recipient), and the relationship between the caregiver and the care-recipient. Also, condition-specific questions were included if the caregiver had a diagnosis of SCD themselves or if the care-recipient had any additional chronic diseases. The survey also included an EQ-5D-5L questionnaire to assess HRQoL, one caregiver-specific questionnaire (CarerQol-7D), and the Work Productivity and Activity Impairment questionnaire: Specific Health Problem (WPAI: SHP), used to assess economic burden.

Caregivers of patients with SCD living in the UK and France were recruited by a market research panel company to complete our cross-sectional survey.

### Inclusion criteria

A sample of caregivers was recruited by a third-party vendor who used existing panels of SCD caregivers and required proof of diagnosis via a doctor’s letter. This type of recruitment allowed caregivers anonymity allowing them to be more open to express themselves freely, that may not happen if recruitment was complete in clinic visits. Our inclusion criteria focused on informal caregivers rather than paid caregivers. Respondents were provided with the following definition of a caregiver: “A caregiver is someone who looks after a family member, partner, or friend who needs assistance because of their illness and cannot manage without their support. The care that caregivers provide is usually unpaid. For example, caregivers will not receive a wage from an employer for providing care.” The respondent had to confirm the definition provided reflected their caregiving circumstance.

The inclusion criteria for caregivers were respondents of age ≥ 18 years who were resident in the UK or France, being the main caregiver for a patient (or multiple patients) clinically diagnosed with SCD by a healthcare professional, could understand English or French, and able to self-complete the online survey. All respondents provided confirmation of informed consent to participate in the study. In addition, because information about patient symptoms was also being collected, caregivers confirmed that their care-recipient(s) verbally consented to information about their diagnosis, treatment, and symptoms being reported. Ethical approval for the study was obtained on March 17, 2022. from an independent ethics reviewer working under the auspices of the Association of Research Managers and Administrators.

### Measures

Each of the measures used in this study were carefully selected for their validity and robustness to ensure the reliability of our findings. These instruments are well-established and routinely used in similar research involving caregivers of individuals with chronic illnesses. This provides a solid foundation for our analysis and conclusions which can be used in cost effectiveness analyses.

### EQ-5D

The EQ-5D-5L is a well-established, generic, preference-based instrument, which has been validated across many disease areas [9]. It assesses your HRQoL on the day of completion across five dimensions (mobility, self-care, usual activities, pain/discomfort, and anxiety/depression), each characterized by five levels of severity (no problems, slight problems, moderate problems, severe problems, extreme problems). Each health profile described by EQ-5D-5L has a utility value on a scale; 1 represents full health and 0 represents a state as bad as being dead. EQ-5D-5L also contains a visual analogue scale (EQ-VAS) that asks respondents to self-report their general health on the day they complete the questionnaire. EQ-VAS ranges from 100 (best health you can imagine) to 0 (worst health you can imagine) [10]. Valuation sets, which are elicited from members of the public, have been generated for many countries, including the UK [11] and France [12].

### Carer Quality of Life 7-Dimension (CarerQol-7D)

The Carer Quality of Life 7-Dimension (CarerQol-7D) is a validated, caregiver-specific instrument for measuring current QoL, consisting of two parts: a descriptive system with seven burden dimensions: two positive dimensions of caregiving (fulfillment and support) and five negative dimensions (relational problems, mental health, physical health, financial problems, and problems combining daily activities with caring), each characterized by three levels (no, some, a lot) [13]. These scores are placed on a scale of 0 (worst informal care situation) to 100 (best informal care situation) using a utility tariff. The available CarerQol-7D utility scores reflect societal preferences for each caregiving state described within the CarerQol-7D descriptive system. The UK tariff related to the CarerQol was applied to data collected from France because a French value set does not currently exist [14]. The CarerQol-7D also contains a general question (CarerQol-VAS) asking respondents to self-report how happy they feel at the time they complete the questionnaire. The CarerQol-VAS ranges from 0 (completely unhappy) to 10 (completely happy) [15].

### Work Productivity and Activity Impairment: Specific Health Problem (WPAI: SHP)

The Work Productivity and Activity Impairment: Specific Health Problem (WPAI: SHP) is a self-administered questionnaire with a 7-day recall period; it was used to elicit from respondents quantified measures of the impact of caregiving on disease on productivity, including absenteeism, presenteeism, work productivity, and activity limitation [16]. The WPAI: SHP consists of six questions to establish the respondent’s employment status, hours missed from work due to the disease or other reasons, number of hours worked, and the extent to which their condition affects both work productivity and non-work-related daily activities. Higher scores suggest greater levels of impairment. WPAI: SHP is constructed so that it can be modified for various needs, and after consultation with the developers of the instrument, a caregiver version for SCD was developed by the study researchers.

### Statistical analysis

The sociodemographic characteristics of caregivers and the patients they care for were summarized using frequencies and means and standard deviations (SD).

Mean (SD) EQ-5D-5L scores were summarized. In line with the updated National Institute for Health and Care Excellence (NICE) guidelines, EQ-5D-5L scores from UK respondents were mapped to the English EQ-5D-3L value set [11]. The EQ-5D-5L scores from the French respondents were converted using the French EQ-5D-5L value set [12]. Pooled scores of all respondents were valued using the English value set. The mapped EQ-5D-5L scores were compared with UK age- and gender-adjusted general population norms. Population norms were calculated using the methodology described by Ara et al., which generates an approximation of the population utility value that the sample would have if they did not have caregiving responsibilities based on the age and gender of our sample [17]. This value was then subtracted from the caregiver utility calculation to generate an age- and gender-matched UK population utility decrement. This decrement value represents the additional reduction in HRQoL that caregivers of individuals with SCD encounter compared with an age- and gender-matched population without caregiving responsibilities.

Based on the WPAI: SHP response data from caregivers, four scores were derived quantifying the impact of their caregiving: percentage of absenteeism; percentage of reduced productivity at work; an overall work impairment score that combines absenteeism and reduced productivity; and percentage of impairment in daily activities performed outside of work [16]. These were summarized using the mean (SD), minimum, and maximum percentages for all the derived scores. Moreover, the total costs per caregiver were calculated using the human capital approach [18], which places a monetary value on the loss of health that equals the lost value of economic productivity due to caregiving. This cost was calculated by multiplying the working hours lost due to caregiving (derived from WPAI: SHP) by either the UK or French hourly minimum wage in 2022, which were estimated at £9.50 [19] and €10.85 [20] respectively. The conservative methodology used in this study assumed that all caregivers received the minimum wage applicable to those aged ≥ 23 in the UK.

An exploratory subgroup analysis was undertaken to assess whether the caregiver had been diagnosed with SCD themselves, as this may be an important factor affecting their QoL.

A sensitivity analysis on productivity costs was undertaken by varying the unit cost attached to wage values and using the average labor wage instead of the minimum wage. The UK average labor wage and the European Union average hourly labor costs in 2019 and 2021 were applied and estimated at £12.17 [21] and €29.01 [22], respectively.

## Results

Sociodemographic characteristics of the caregivers included in this study are described in Table 1. A total of 69 caregivers (for 81 patients with SCD) were recruited, of which 43 resided in the UK and 26 resided in France.

**Table 1:** Sociodemographic characteristics of caregivers

Characteristics of caregivers^a^	UK (*n* = 43)	France (*n* = 26)	Pooled (*N* = 69)
Number of patients with SCD cared for			
1	38 (88)	20 (77)	58 (84)
2	5 (12)	5 (19)	10 (14)
3	0 (0)	1 (4)	1 (2)
Age in years			
18‒34	5 (12)	14 (54)	19 (28)
35‒54	33 (76)	11(42)	44 (64)
55+	5 (12)	1 (4)	6 (9)
Gender			
Female	36 (84)	21 (81)	57 (83)
Caregiver has SCD			
Yes	10 (23)	5 (19)	15 (22)

*SCD* sickle cell disease, *UK* United Kingdom, *N* total sample, *n* subsample

^a^All data are *n* (%). Percentages may not add to 100% due to rounding

Table 2 describes the sociodemographic characteristics of patients with SCD, as reported by their caregivers. Most patients were aged 4‒17 years and female. Caregivers were most likely to be the parent of a patient with SCD.

**Table 2:** Sociodemographic characteristics of patients with SCD^a^

Characteristics of patients with SCD	UK (*n* = 48)	France (*n* = 33)	Pooled (*N* = 81)
Age in years			
0‒3	6 (23)	5 (15)	11 (14)
4‒11	11 (13)	11 (33)	22 (27)
12‒17	15 (31)	7 (22)	22 (27)
18‒34	10 (21)	6 (18)	16 (20)
35‒54	3 (6)	4 (12)	7 (9)
55+	3 (6)	0 (0)	3 (3)
Gender			
Female	26 (54)	17 (52)	43 (53)
Impact of SCD			
A big impact	22 (46)	8 (24)	30 (37)
A noticeable impact	13 (27)	14 (42)	27 (33)
Somewhat of an impact	10 (21)	7 (21)	17 (21)
Very little impact	2 (4)	4 (12)	6 (7)
No impact at all	1 (2)	0 (0)	1 (1)
Other health condition besides SCD			
Yes	20 (41)	11 (33)	31 (38)
Relationship			
Parent	33 (69)	16 (48)	49 (60)
Child	5 (10)	8 (24)	13 (16)
Other	10 (21)	9 (27)	19 (23)

*SCD* sickle cell disease, *N* total sample, *n* subsample

^a^All data are n (%)

Overall, the mean (SD) EQ-5D-5L utility score for caregivers was 0.66 (0.28) (UK tariff, pooled sample). The average EQ-VAS score was 68.67 and ranged from 10 to 100. Lower EQ-5D scores were observed in the UK sample using both the NICE and the French value sets (Table 3). Examining the caregivers utility scores compared with age- and gender-matched general population utility norms [17], it was determined that based on an approximated population utility norm of 0.89, caregivers of people with SCD had a utility decrement of between 0.11 and 0.23 (adjusted for age and gender), as measured using the EQ-5D-5L.

**Table 3:** EQ-5D-5L results

	UK tariff		French tariff	
	Mean (SD)	95% CI	Mean (SD)	95% CI
EQ-5D-5L^a^				
Pooled (*N* = 69)	0.66 (0.28)	0.59‒0.73	0.78 (0.31)	0.70‒0.85
UK only (*n* = 43)	0.62 (0.29)	0.53‒0. 71	0.74 (0.34)	0.63‒0.84
French only (*n* = 26)	0.73 (0.24)	0.63‒0.82	0.84 (0.24)	0.74‒0.94
	**Mean (SD)**	**Range**		
VAS^a^				
Pooled (*N* = 69)	68.67 (21.98)	10‒100		
UK only (*n* = 43)	70.30 (21.66)	15‒100		
French only (*n* = 26)	65.96 (22.67)	10‒100		

	**UK tariff**,** pooled sample (*N*** **= 69)**		**French tariff**,** pooled sample (*N*** **= 69)**	
Age- and gender-matched EQ-5D-5L difference in utility score decrement^b^	0.23		0.11	

	EQ-5D-5L UK tariff		**VAS (UK)**	
	**Mean (SD)**	**95% CI**	**Mean (SD)**	**Range**
Caregiver subgroup analysis				
Has SCD (*n* = 15)	0.62 (0.23)	0.49‒0.75	65.86 (24.57)	33‒100
Does not have SCD (*n* = 52)	0.67 (0.53)	0.59‒0.76	69.60 (21.66)	10‒100

*CI* confidence interval, *NA* not applicable, *SCD* sickle cell disease, *SD* standard deviation *VAS* visual analogue scale, *N* sample, *n* subsample

^a^By population sampled

^b^Decrement based on the difference between population age- and gender-matched utility scores in the absence of caregiving responsibilities (0.89 in UK) and the presence of caregiving responsibilities for SCD (0.66 in the UK tariff; 0.78 in the French tariff)

Overall, caregivers reported a mean (SD) CarerQol-7D utility score of 80.69 (24.40) (pooled sample). The average CarerQol-VAS was 6.23 (pooled sample) and ranged from 1 to 10. Lower utility and VAS scores were observed in the UK (Table 4).

**Table 4:** CarerQol utility results

CarerQol^a^	Mean (SD) UK tariff,	95% CI
Pooled (*N* = 69)	80.69 (24.40)	74.83‒86.56
UK only (*n* = 43)	78.72 (25.79)	70.78‒86.65
French only (*n* = 26)	83.97 (22.01)	75.07‒92.86

**CarerQol-VAS** ^**a**^	**Mean (SD)**	**Range**
Pooled (*N* = 69)	6.23 (2.15)	1‒10
UK only (*n* = 43)	6.19 (2.29)	2‒10
French only (*n* = 26)	6.31 (1.93)	1‒10

**Caregiver subgroup analysis—CarerQol-7D**	**Mean (SD)**	**95% CI**
Has SCD (*n* = 15)	71.87 (23.13)	59.06‒84.68
Does not have SCD (*n* = 52)	83.90 (24.40)	77.11‒90.69

**Caregiver subgroup analysis—CarerQol-VAS**	**Mean (SD)**	**Range**
Has SCD (*n* = 15)	6.73 (2.52)	3‒10
Does not have SCD (*n* = 52)	6.12 (2.074)	1‒10

*CarerQol-7D* Carer Quality of Life 7-Dimension, *SCD* sickle cell disease, *SD* standard deviation, *UK* United Kingdom, *VAS* visual analogue scale, *N* sample, *n* subsample

^a^Results by population sampled

Exploratory subgroup analysis was undertaken for the EQ-5D-5L results and the CarerQol-7D for the groups of caregivers with and without SCD. Respondents who answered “don’t know” to the question about whether they had a diagnosis of SCD were excluded from the analysis. The EQ-5D-5L results determined that there was a 0.06 decrement for caregivers with SCD compared with caregivers without SCD, and a decrement of 15.25 between the subgroups was also identified in the CarerQol-7D.

**Fig. 1:**
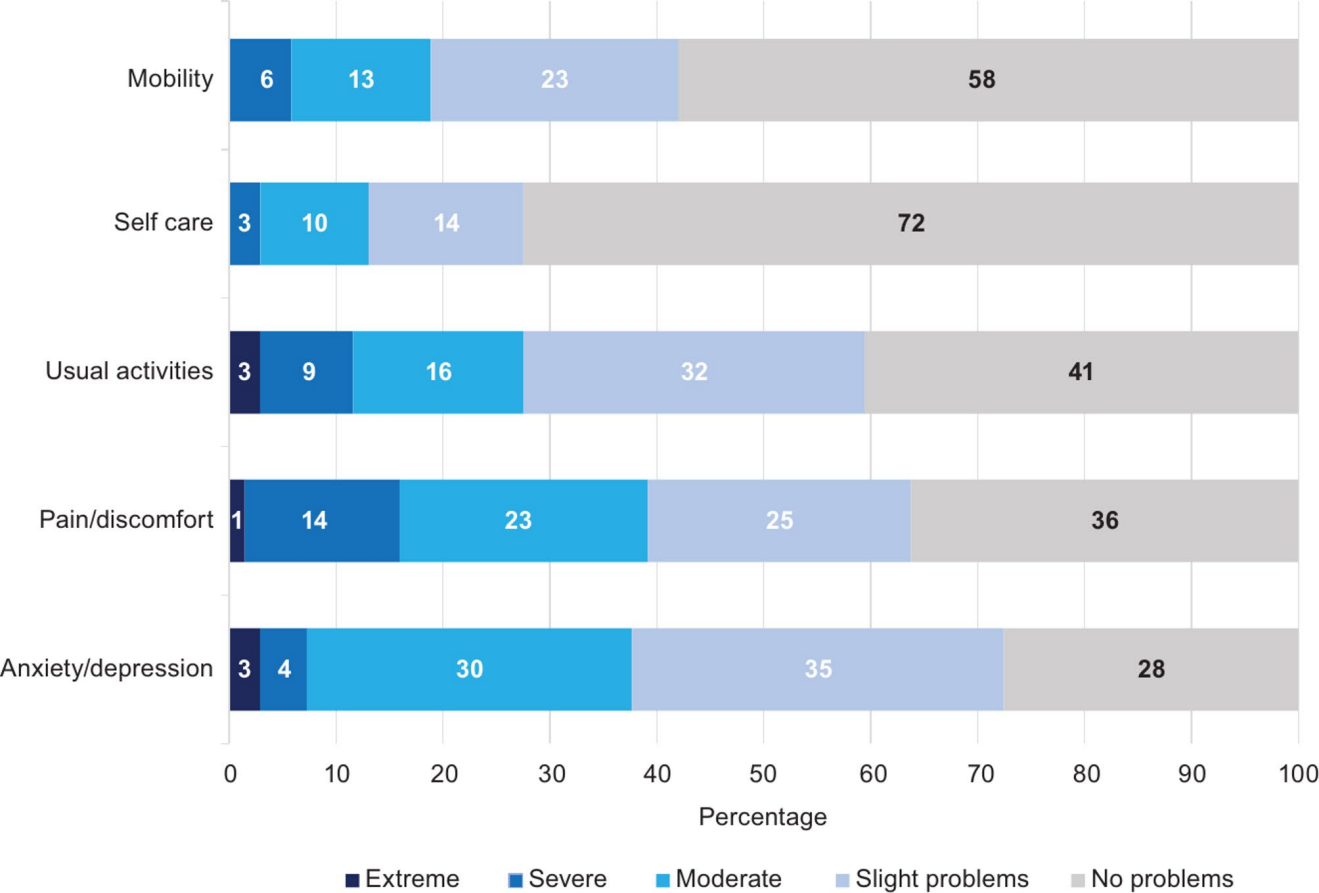
EQ-5D-5L dimensions for caregivers (pooled data)

**Fig. 2:**
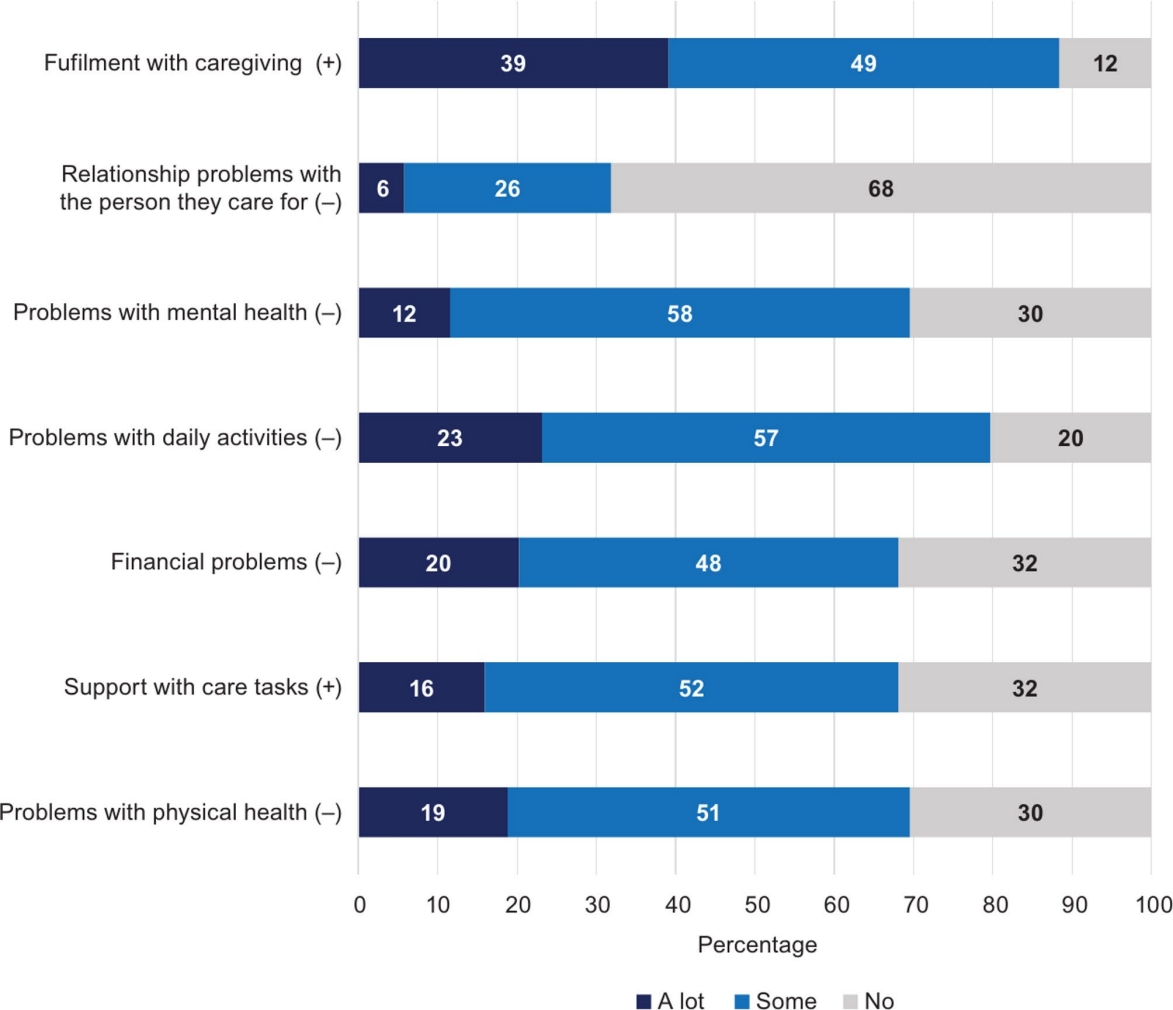
CarerQol-7D dimensions for caregivers (pooled data) ((+) indicates the positive aspects of caring (“a lot” = best level); (‒) indicates the negative aspects of caring (“a lot” = worst level))

Figures 1 and 2 provide further granularity regarding the responses to the EQ-5D-5L and CarerQol-7D by burden dimensions, respectively. Figure 1 shows that respondents reported more variation in their domain level choices for the anxiety/depression dimension. A high proportion of caregivers had slight (35%) and moderate problems (30%) for the anxiety/depression dimension. No participant reported extreme problems in the mobility or self-care dimension.

**Fig. 3:**
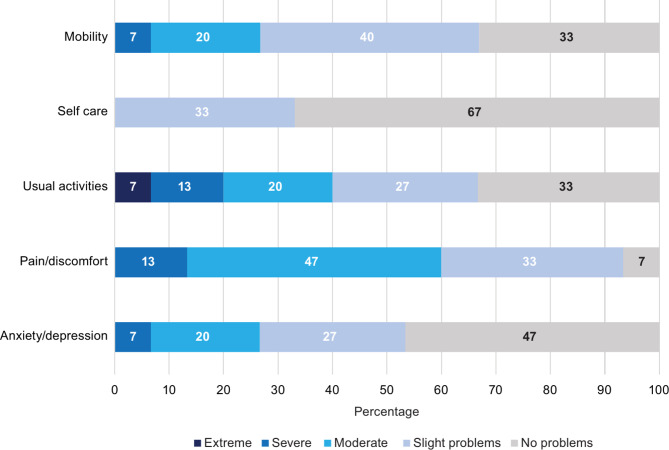
EQ-5D-5L dimensions for caregivers with SCD

**Fig. 4:**
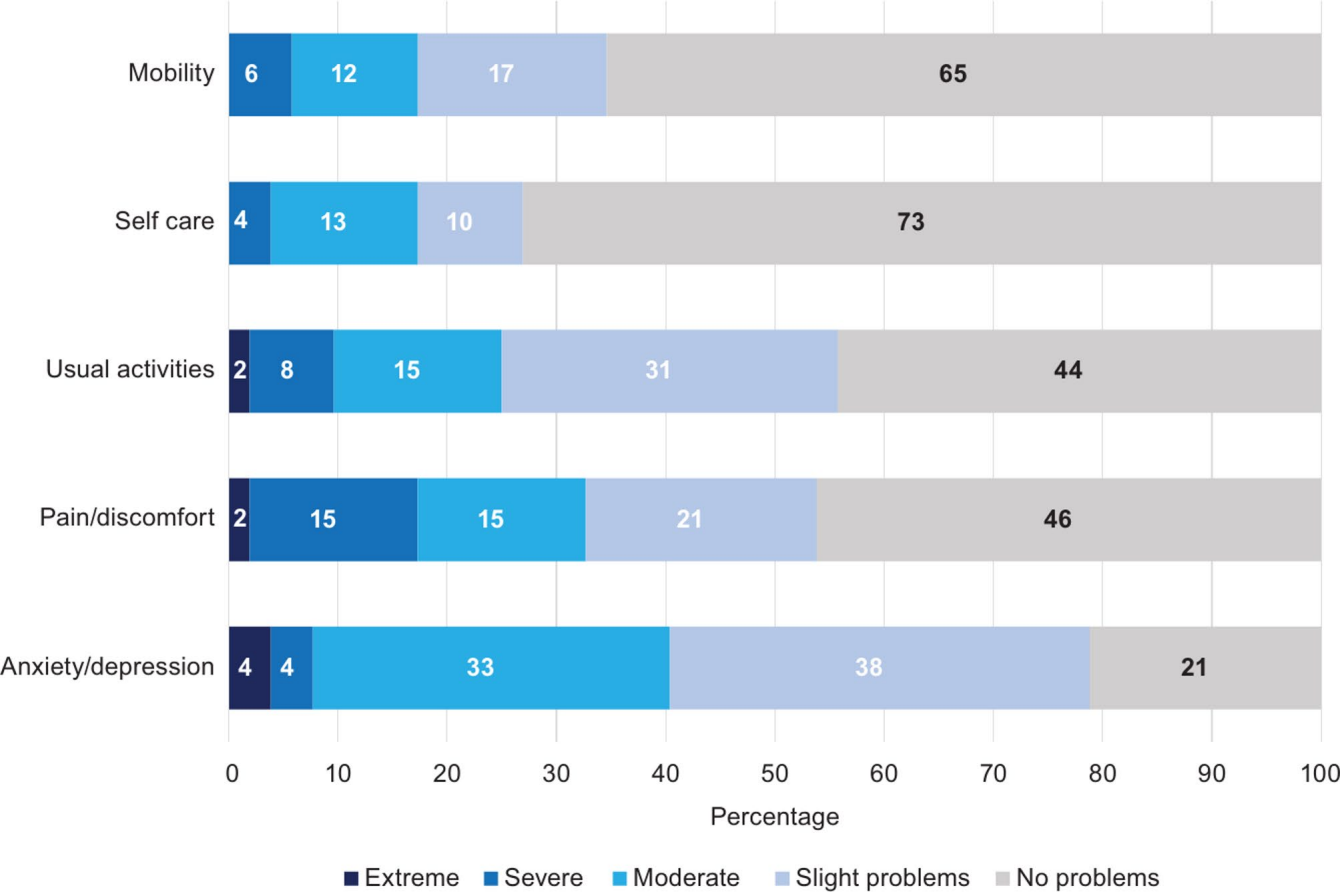
EQ-5D-5L dimensions for caregivers without SCD

Similarly, in the CarerQol-7D measures, 64% of respondents indicated that they experience at least some mental health issues (Fig. 2). Figures 3 and 4 examine the EQ-5D-5L domain values of caregivers with and without SCD, respectively. In the pain/discomfort domain, 60% of caregivers with SCD reported moderate to severe problems compared with 30% of the caregivers without SCD. Among caregivers, 93% with SCD reported some degree of pain/discomfort compared with 53% without SCD. Anxiety/depression was observed to be lower in those with SCD than those without SCD. However, the mobility, self-care, and usual activities domain findings indicated worse problems for caregivers with SCD than without. Further analysis completed on the CareQol-7D measure comparing those with and without SCD is reported in the supplementary material.

Table 5 shows the WPAI: SHP results of caregivers in employment. Thirty-seven caregivers were employed, and they reported missing an average of 22% of work hours due to their caregiving responsibilities. All caregivers responded about the impact of caregiving on their ability to undertake normal daily activities; on average 53% reported that caregiving affected their ability to undertake normal daily activities.

**Table 5:** WPAI: SHP results: mean percentage of absenteeism, presenteeism, work productivity, and activity limitation among caregivers

WPAI: SHP metric (score)	*n*	Mean (SD) %	Minimum	Maximum
Absenteeism (1)—work time missed due to caregiver burden (past 7 days)	37	22 (24)	0	100
Presenteeism (2)—impairment while working due to caregiver burden (past 7 days)	37	46 (24)	0	100
Work productivity (3)—overall work impairment due to caregiver burden	37	34 (19)	0	100
Activity limitation (4)—impairment in daily activities due to caregiver burden (past 7 days)	69	53 (24)	0	100

*n* subsample, *SD* standard deviation, *WPAI: SHP* Work Productivity and Activity Impairment Questionnaire: Specific Health Problem

Table 6 reports the annual cost of the lost work hours because of caregiving. All the hours lost annually were valued using the minimum wage of the caregivers’ respective country of residence. Lost productivity per week per employed caregiver was estimated as £4209 in the UK and €3485 in France annually.

**Table 6:** Caregiver productivity loss by country

Base case	*n*	Annual mean economic costs
UK	24	£4209
France	13	€3485

Sensitivity analysis	n	Annual mean economic loss
UK	24	£5391
France	13	€9319

*UK* United Kingdom, *n* subsample

## Discussion

In this cross-sectional, multinational study, we assessed the effects of caregiving on the HRQoL, economic burden, and productivity, of carers providing support to patients with SCD using validated survey instruments. Caregiving was found to have a negative impact on the HRQoL of caregivers’ and caused a large financial and social burden.

Examining the differences in HRQoL between the two countries, higher HRQoL ratings among French caregivers may be related to their younger age profile (54% aged 18–34 vs. 12% in the UK), as younger caregivers may have a more positive approach to caregiving and fewer health related co-morbidities [23]. Additionally, UK caregivers in this sample might have faced more challenges, such as caring for young people aged 12–17 during a critical developmental period (31% in the UK vs. 22% in France), a higher proportion of carers had SCD themselves, and being an older cohort had the challenge of managing both age and SCD associated co-morbidities.

Comparing the findings of our study with others is challenging because of the significant variation among countries regarding social values (i.e., social expectations, pressure, hierarchy, and cultural family dynamics) and economic supports for caregivers which allow them to function beyond their caregiving role. However, similar findings from previous studies indicate emotional distress [24] and depressive moods [25, 26] among caregivers of people with SCD. The present study reported that 68% of caregivers had at least some issues with their mental health. Another multinational study (SHAPE) investigating the burden of SCD found that the mental health of 52% of caregivers was impacted, although the SHAPE study did not use validated measures [27]. Of the caregivers in this study, 80% reported at least some problems within the daily activities dimension in the CarerQol-7D. A similar trend was observed in caregivers from the Netherlands; caregivers of children with SCD demonstrated significantly lower QoL scores on the Netherlands Organisation for Applied Scientific Research Academic Medical Centre, or TNO-AZL Adult Quality of Life questionnaire subscale for daily activities [26]. “Relationship problems” was one of the least impacted CarerQol-7D domains in our study, with 68% of caregivers reporting no issues. Similar results were observed in previous research; 70.7% of caregivers reported no problems being faced in family interactions in one study [28], and another study revealed that family life dynamics were the least stressful aspect of being a caregiver to a person with SCD [29]. Further examination of caregiver burden utilities (collected using the CarerQol-7D utility scores) in cystic fibrosis, which is also an inherited, life-threatening disease, reported a similarly high mean utility of 84.6 [30]. In addition, the mean (SD) EQ-5D-5L utility score—of 0.62 (0.26) in this study mapped to the EQ-5D-3L version—was found to be more similar to the score for adults with SCD—of 0.65 (0.29)—who were being discharged after a hospital stay than the same sample 7 days after discharge from the hospital—0.75; SD, 0.26—as measured using the EQ-5D-3L UK tariff [31]. This comparison between caregivers and patients with SCD highlights the significant caregiver burden that caregivers with SCD experience.

Furthermore, SCD has a very high allele prevalence in the at-risk population, which is why it affects additional family members more frequently than in other genetic diseases. Thus, this study identified a higher prevalence of caregivers with SCD in the sample compared with other genetic diseases. This study showed there is a lower HRQoL for caregivers with SCD who experience higher levels of pain/discomfort and greater impacts on everyday life. This may highlight an unmet need for this vulnerable caregiver group, as caregivers in this sample may focus on the health and emotional needs of the person with SCD and neglect their own. This, together with managing their own SCD symptoms, may have a significant compounding effect on the HRQoL of caregivers with SCD. The pain/discomfort reported by caregivers without SCD was higher than expected. We postulate that pain may occur due to physically and emotionally supporting patients. Complications such as stroke, avascular necrosis, debilitating pain episodes, and mobility impairment, etc. can impose increased physical demands on assisting caregivers leading to musculoskeletal discomfort associated with caring, as has been the case in other caregiver populations [32].

We estimated high productivity costs measured using the WPAI: SHP. In addition, the WPAI: SHP indicated higher percentages of absenteeism, presenteeism, impaired work productivity, and activity limitation due to caregiver burden. While there is little evidence of the productivity burden on caregivers, a multinational study (SWAY) that examined the working population with SCD found a similar pattern—patients with SCD had missed an average of seven hours of work over the previous seven days [33]. Our study highlights the greater impact of caregiving on productivity, particularly in the case of UK caregivers, who were estimated to miss 2.2 more hours of work than patients with SCD in the SWAY study. Studies examining the financial burden of SCD on relatives of patients further confirmed our findings that family members experienced significantly greater financial burden [28, 34, 35]. The SHAPE study found over half (56%) of caregivers were impacted in their ability to attend and succeed at school or work due to their caregiving duties [36]. This was further confirmed in a cross-sectional study at a tertiary care center in Saudi Arabia, in which the estimated cost of caregiving was high relative to the socioeconomic status of the participants’ families [25]. Furthermore, this impacted the HRQoL of caregivers so that, of the 13 life domains measured, the financial situation domain received the lowest satisfaction score [25]. Our study found that 68% of caregivers reported at least some financial problems in this dimension of the CarerQol-7D; similarly, in the UK sample of the SHAPE survey, 57% of respondents felt that caring for someone with SCD impacted their earning potential [36]. These results align with our study results and confirm that responsibilities of caring for patients with SCD are associated with substantial financial implications for caregivers.

Although increasing research has been conducted on the HRQoL of patients with SCD, the HRQoL of their caregivers has not been examined comprehensively using validated standardized tools. This study describes the use of two validated HRQoL instruments—the generic EQ-5D-5L and the carer-specific CarerQol-7D. The EQ-5D-5L allows us to compare the HRQoL of caregivers between the general population, patients, and also allows for a HRQoL comparison across caregivers who care for patients with different diseases [9]. The CarerQol-7D assesses both positive and negative aspects of providing care, which paints a more complete picture; it includes the health dimensions specific to the caregivers’ situation (e.g., fulfillment), which may have been missed using the EQ-5D-5L [15]. The use of the WPAI: SHP allows for a separate estimation of work time missed, impairment at work, and effect on daily activities, thus expanding our knowledge of the impact of caregiving on caregivers with SCD [16]. These results can provide evidence of caregiving-related costs incurred by both employers and society. We also conducted sensitivity and subgroup analysis to test our hypothesis and explore the impact of caregiving for people with SCD on both HRQoL and productivity.

This study has some limitations. Considering the cross-sectional nature of our survey, it is important to note that respondents were at different stages of their caregiving journey. Our study included caregivers of both children and adults with SCD, reflecting diverse caregiving experiences across different life stages and for varying durations. Because this was not longitudinal study, we were unable to determine whether the impact of caregiving duties on QoL may be something longer-term caregivers adapt to over time, but which those relatively new to caregiving duties find more onerous. Despite this, the variability did allow us to capture a broad spectrum of perspectives and challenges faced by caregivers throughout the lifespans of individuals affected by SCD. By encompassing caregivers at various life stages, a comprehensive picture is provided of the impact of caring for people with SCD.

In future research, some additional considerations based on patients’ disease severity may need to be factored in when measuring caregiver burden (including age of the person with SCD, number of hospital admissions, number of acute complications, existence of long-term complications, or when treatment was received). These additional considerations—not included in the analysis—may have impacted the lifestyle of caregivers, which may have affected their evaluation of HRQoL and the results from respondents. Second, caregivers who are significantly impacted by caregiving duties may not have participated in the study. Caregivers with less opportunity and time may have been missed from our sample despite our best efforts to optimize the speed at which the survey could be completed while also collecting the most pertinent data. In addition, the recruitment of caregivers via a patient group or panel could have influenced the results of the study; it is possible that those caregivers may have experienced more care-related difficulties, and therefore seek help through patient groups, thus introducing a possibility of bias in our results. Another consideration is the use of the UK tariff for the CarerQol values from the French sample. Because tariffs are often developed to account for specific cultural, economic, and healthcare contexts, they can differ significantly between countries. This discrepancy can affect the relevance and applicability of the results as the population may perceive and value health states differently. Finally, for productivity costs, the monetary value associated with caregiving-related productivity loss at baseline was estimated using the average minimum wage. This is likely to be a conservative estimate, as the most common caregiver age category in the study was 35‒54 years, an age range typically inclusive of peak earning potential [37]. Hence, the additional sensitivity analysis was performed.

The definition of caregivers in our survey focused on informal caregivers and excluded professional caregivers. This exclusion criteria may have inadvertently excluded caregivers who are receiving an allowance from the government for caregiving activities and who may have believed that they were not eligible for inclusion in the study. The definition of informal caregivers has been previously used across other caregiver research without detecting any issues [38]. Nevertheless, this may have impacted recruitment into the study. The study sample was also difficult to recruit as some caregivers may feel stigma associated with the disease due to negative social attitudes [39].

This study provided insight into the complex burden of caring for patients with SCD by surveying caregivers about their HRQoL as well as economic and clinical burdens. Caregivers contribute significantly to maintaining and improving the HRQoL of patients with SCD [26]. Despite this, unlike some diseases, the caregiver burden in SCD can go largely unnoticed [40]. In addition, it is likely that a significant proportion of caregivers have a diagnosis of SCD themselves, and/or care for more than one patient with SCD within a family unit, as found in this study [41]. As such, there is an additional stress on health as well as financial and educational access for families, thereby perpetuating inequalities across generations of families living with SCD [42]. It is important to highlight this additional burden to generate evidence about the impact of caregiving responsibilities on caregivers and increase awareness and support for them within health systems. While the medical management of patients with SCD is important, so too is the well-being of their caregivers, and this is an area deserving of significant attention. Improvement in the health of the caregiver would likely enable better care of patients with SCD. When determining the kind of support caregivers of people with SCD require, undertaking a comprehensive needs assessment can inform interventions for caregivers and help prioritize resources to address key deficiencies in healthcare services. To do this, we need a better understanding of the impact of different phases of support required for patients with varying degrees of SCD severity, particularly based on their age or number of SCD crises, as it may shift the type of support caregivers with SCD require. These shifts may assist caregivers in processing and managing the impact of SCD on themselves and possibly highlight additional caregiver needs. Caregiver interventions that improve knowledge about navigating health and social care systems [43, 44] and aid in the access of better support may mitigate the psychosocial burden on caregivers of people with SCD [45].

The effects of patient outcomes or disease trajectory on caregivers as well as the origin of their reported domains is currently unknown. Thus, further research on the HRQoL of caregivers of patients with SCD, including further development of the study and expansion of the current study to other countries and qualitative studies to identify the origins of the domains’ answers, would be beneficial to validate our study findings. Additionally, a longitudinal survey that links patients with SCD and their caregivers could help to identify the potential unmet needs required to optimize support. Furthermore, a longitudinal study of this nature would increase the robustness of our study results and could help with an examination of the relationship between long-term levels of disability and outcomes such as HRQoL, productivity, and burden on these caregivers.

## Conclusion

Caring for patients with SCD has a negative impact on caregivers’ HRQoL and work productivity. Caregivers play an important role in the lives of the individuals with SCD that they care for. Further support and intervention can be crucial to meeting the complex needs of caregivers and alleviating their economic burden.

## Electronic supplementary material

Below is the link to the electronic supplementary material.

Supplementary Material 1

## Data Availability

Our data cannot be shared openly in the interests of protecting the privacy of study participants.

## Abbreviations

CarerQol-7D: Carer Quality of Life 7-Dimension
EQ-VAS: EuroQol-visual analogue scale
HRQoL: Health-related quality of life
NICE: National Institute for Health and Care Excellence
QoL: Quality of life
RBC: Red blood cells
SCD: Sickle cell disease
SD: Standard deviation
UK: United Kingdom
VOC: Vaso-occlusive crisis
WPAI:SHP: Work Productivity and Activity Impairment Questionnaire: Specific Health Problem
